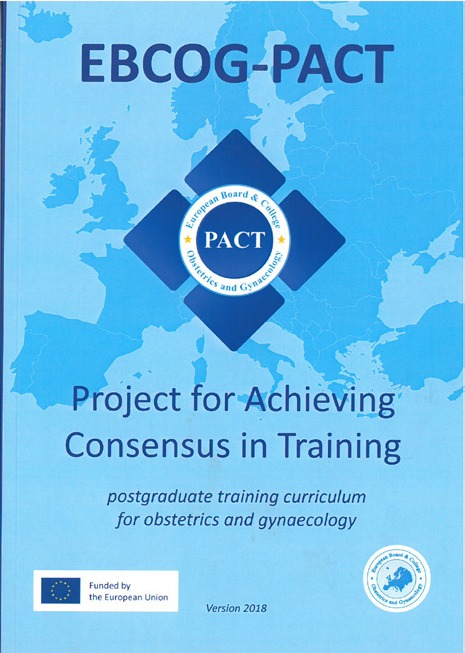# Improving the training of the future gynaecologist: development of a European curriculum in Obstetrics and Gynaecology (EBCOG-PACT)

**Published:** 2018-03

**Authors:** JE Van der Aa, AJ Goverde, F Scheele

**Affiliations:** Department of Research and Education, OLVG Hospital, Amsterdam, the Netherlands; Athena Institute, Faculty of Science, VU, Amsterdam, the Netherlands; European Board & College of Obstetrics and Gynaecology, Brussels, Belgium; Department of Reproductive Medicine and Gynaecology, University Medical Centre, Utrecht, the Netherlands; Department of Obstetrics and Gynecology, Amsterdam UMC, VU University, Amsterdam, the Netherlands

**Keywords:** PGME, training, harmonisation, Europe, EBCOG, PACT, Obstetrics and Gynaecology

## Abstract

The European Board & College of Obstetrics and Gynaecology has initiated improvement of the European standards of training in Obstetrics and Gynaecology through the project called ‘EBCOG-PACT’. In this project, a pan-European curriculum for postgraduate training in Obstetrics and Gynaecology has been developed. The curriculum is societally responsive, and based on the latest medical educational methodology. It consists of the description of outcomes of training for the common Core Curriculum and Electives, the General competencies and soft skills to be trained, and strategies for training of obstetrical skills, gynaecological skills, ultrasound skills and bio-psychosocial and communicative skills. Also, the curriculum provides strategies for assessment through entrustment, a model for portfolio as well as strategies for faculty development and quality management of training. The implementation of the European curriculum in Obstetrics and Gynaecology will provide opportunities for national scientific and professional societies and ministries of health or education to consider modernisation of national or local OBGYN training programs.

In the ever more internationalising world of today, with increasing mobility of both patients and doctors, training of the gynaecologist of the future requires high quality curricula that allow for continuous improvement and modernisation. Therefore, the European Board & College of Obstetrics and Gynaecology (EBCOG) has taken up the task to initiate improvement of the European standards of training in Obstetrics and Gynaecology (OBGYN) through the project called ‘EBCOG-PACT’ (Project for Achieving Consensus in Training). In this project, a pan-European curriculum for postgraduate training in OBGYN has been developed, which is societally responsive, and based on the latest medical educational methodology.

Three main reasons clarify why development of a pan-European curriculum in OBGYN, and therefore European harmonisation of training, is needed (van der Aa at al., 2016). Primarily, the implementation of high quality standards of women’s healthcare will be enhanced by the implementation of a pan-European curriculum, ensuring high quality training of gynaecologists (EBCOG standards of care 2014-gyn; EBCOG standards of care 2014-obs). Secondly, quality of training in OBGYN will be assured for trainees, gynaecologists and hospitals throughout Europe, and therefore mobility of trainees and gynaecologists in Europe will be promoted. Thirdly, cooperation and exchange of best practices between medical specialists and hospitals will be further enhanced, allowing for improvement of communication and collaboration between gynaecologists and hospitals to deliver high quality care to the mobile patients in Europe (van der Aa at al., 2016).

The PACT project has been executed in close collaboration between European gynaecologists, trainees, educationalists, change managers, midwives, specialized nurses, hospital board members, and last but not least, patient representatives. It was recognised as a valuable strategic partnership for improvement of European higher education by the European Commission, and therefore was awarded an ‘Erasmusplus’ grant. Partners in this project represented four highly-renowned medical institutions from Denmark, Italy, Czech Republic, Belgium and the Netherlands, as well as organisations representing the societal stakeholders.

The project ran between September 2015 and September 2018 and has resulted in several outcomes. The curriculum consists of the description of outcomes of training for the common Core Curriculum and Electives, both developed through a (Delphi) consensus procedure amongst European gynaecologists and trainees (van der Aa at al., 2017). Besides the medical competencies, the curriculum also describes the ‘General competencies and soft skills’ developed by representatives of European patients, midwives, specialised nurses, hospital board members, trainees and gynaecologists. Experts developed and described a wide range of strategies for training of obstetrical skills, gynaecological skills, ultrasound skills and bio-psychosocial & communicative skills. Also, the curriculum provides strategies for assessment through entrustment, a model for portfolio as well as strategies for faculty development and quality management of training. In the final stage of the project a SWOT-analysis was carried out, giving input for recommendations for sustainable implementation of the curriculum in Europe. A handbook was developed to advise those embarking on a similar project to harmonise postgraduate training.

The curriculum has been translated and is currently available in seven different languages (English, Spanish, German, Italian, Polish, Russian, Portuguese) with two additional languages soon to follow (French, Czech). Also, several national scientific societies have initiated translation of the curriculum in their national language (Greece, Slovenia, Turkey).

The UEMS council has officially adopted the curriculum as the European Training Requirements (ETR) in the field of Obstetrics and Gynaecology (https://www.uems.eu/areas-of-expertise/postgraduate-training/european-standards-in-medical-training). We are convinced that the implementation of the European curriculum in OBGYN will provide opportunities for national scientific and professional societies or ministries of health and education to consider modernisation of national or local OBGYN training programs.

**Figure:**